# Coping with access barriers to non‐communicable disease medicines: qualitative patient interviews in eight counties in Kenya

**DOI:** 10.1186/s12913-021-06433-0

**Published:** 2021-05-03

**Authors:** Gloria Ng, Elizabeth Raskin, Veronika J. Wirtz, Kathleen P. Banks, Richard O. Laing, Zana W. Kiragu, Peter C. Rockers, Monica A. Onyango

**Affiliations:** 1 Department of Global Health, Boston University School of Public Health, Crosstown, 3rd floor, 801 Massachusetts Avenue, MA 02118 Boston, USA; 2 School of Public Health, Faculty of Community and Health Sciences, University of Western Cape, Cape Town, South Africa

## Abstract

**Background:**

There is rich literature on barriers to medicines access for the treatment of non-communicable diseases (NCDs) in high-income countries. Less is known about low- and middle-income countries, in particular the differences in coping with medicines access barrier by household wealth and disease. The aim of this study was to compare the coping mechanisms of patients with the lack of availability and affordability of cardio-vascular diseases, diabetes and asthma medicines in Kenya.

**Methods:**

This qualitative study was part of a larger mixed methods evaluation study conducted in eight counties of Kenya from 2016 to 2019. Forty-nine patient interviews at study end line explored their NCD journey, perceptions of availability, stockouts and affordability of NCD medicines, their enrollment in health insurance, and their relationship with the private chemists. Transcribed interviews were coded using Nvivo software. A two-step thematic approach was used, first conducting a priority coding which was followed by coding emerging and divergent themes.

**Results:**

Overall, we found that patients across all disease types and wealth level faced frequent medicine stock-outs at health facilities. In the absence of NCD medicines at health facilities, patients coped by purchasing medicines from local chemists, switching health facilities, requesting a different prescription, admitting oneself to an inpatient facility, establishing connections with local staff to receive notifications of medicine stock, stocking up on medicines, utilizing social capital to retrieve medicines from larger cities and obtaining funds from a network of friends and family. Categorizing by disease revealed patterns in coping choices that were based on the course of the disease, severity of the symptoms and the direct and indirect costs incurred as a result of stockouts of NCD medicines. Categorizing by wealth highlight differences in households’ capacity to cope with the unavailability and unaffordability of NCD medicines.

**Conclusions:**

The type of coping strategies to access barriers differ by NCD and wealth group. Although Kenya has made important strides to address NCD medicines access challenges, prioritizing enrollment of low wealth households in county health insurance programs and ensuring continuous availability of essential NCD medicines at public health facilities close to the patient homes could improve access.

## Introduction

More than 85 % of premature deaths caused by non-communicable diseases (NCD) occur in low and middle income countries (LMICs) [1]. NCDs can lead to lifelong disability or premature death without adequate treatment using essential medicines. Such uninterrupted patient access to essential medicines is a priority for reducing the NCD burden in any LMIC. In Kenya, the growing prevalence of NCDs accounts for approximately 27 % of all deaths annually [2]. Cardiovascular disease, diabetes, and asthma are among the most prevalent NCDs in Kenya [3, 4].

The Kenya government has made significant strides in addressing the rising NCD burden in the country. A number of policy interventions have been instituted to move towards universal health coverage (UHC) at the national and county levels [5], including national health insurance schemes and scaling up screening, surveillance, health promotion and the capacity of NCD service delivery and referrals [6]. However, the access barriers to NCD medicines in Kenya remain a challenge and have been well documented [7–12]. Medicine shortages and high prices remain the largest access barrier for patients, despite recent policy interventions [5, 6, 10, 11, 13]. Patients from other LMICs have coped with access barriers, and employed strategies such as borrowing funds, selling assets, taking less than the prescribed dose, and relying on social networks to borrow money for medicines [7, 12, 14–19]. However, there is poor understanding of the ways in which NCD patients in certain wealth levels and those with specific diseases attempt to cope with access barriers.

The primary aims of this study were to understand how patients with different levels of wealth and diseases cope with lack of availability and affordability of medicines, as well as any tradeoffs made in the absence of medicines at the household level. Evidence on patient coping strategies can inform policy approaches to reducing the burden of NCDs in Kenya.

## Methods

This qualitative study was part of a larger mixed methods evaluation conducted in eight counties in Kenya from 2016 to 2019. The eight counties included in the study were Embu, Kakamega, Kwale, Makueni, Narok, Nyeri, Samburu, and West Pokot. Data were collected at baseline, midline and end line. The main purpose was to evaluate the impact of Novartis Access Project on the availability and price of NCD medicines included in the program portfolio and their therapeutic equivalents [20]. The first country where the program was roll-out was Kenya. The results of the evaluation are reported elsewhere [21]. This study focus on the interviews conducted with respondents at the end line in 2019.

### Sampling and selection of study participants

The sampling and selection of study participants for the larger study have been described in detail elsewhere [20, 21]. For the purpose of this paper we will describe the sampling that is relevant to this study: the eight study counties were selected based on their security, safety and volume of purchasing medicines from Mission for Essential Drugs and Supplies (MEDS), an organization that supplies medicines to a large network of public and private (often-faith-based) health facilities. NCD services are provided in the public and private non-profit sectors at all levels of the health system.

A two-stage sampling procedure was used: first, ten enumeration areas (EAs), the smallest administrative unit in a county, were established and second, ten eligible households were identified for every enumeration area. The goal was to enroll 800 households, 100 per county. Eligibly households had to have at least one member 18 years or older who had previously been diagnosed and prescribed medicine for either diabetes, asthma, hypertension or breast cancer. If there were more than one member, the other adult members in the household who met the eligibility criteria were also invited to participate. For qualitative analysis, every fifth house in an EA was eligible for an interview after completion of the quantitative surveys. At baseline where we interviewed 84 participants. At endline we interviewed the same participants, but due to loss to follow up we interviewed a total of 49 out of the initial 84.

Prior to data collection during endline evaluation, a two-day qualitative interviewing training was conducted for all enumerators involved in data collection and management. Training was provided on human subject’s protection, research ethics, and qualitative methods. Semi-structured study guides were pre-tested by the study team and developed in English. The research assistants were multi-lingual and most came from the study locations and were able to do simultaneous translation when necessary. The endline study questions explored participants’ NCD journey; perceptions of availability, stockouts and affordability of NCD medicines; participants’ enrollment in health insurance; and their relationship with the private pharmacy and drug sellers. The duration of the interviews ranged from 17 to 46 min.

### Data managements and analysis

Since the study participants spoke, English or Kiswahili and a local language that interviews were transcribed and, when necessary, translated from Kiswahili or local language to English. Two researchers read the typed interview transcripts separately for accuracy and completeness. Transcripts were imported into NVivo 12 qualitative data analysis software for coding and analysis. We used *a priori* coding based on the aims of (or categories related to) the evaluation, then coded for emergent and divergent themes. This was followed by constructing wealth indices and applied them across the themes to further understand the context. The two researchers did the initial coding individually and then compared the codes of emerging themes to reach a consensus, with input from and reflections with three study authors. New codes were created, dropped and and/or combined as appropriate. During the coding process, data was continuously reviewed, emerging patterns noted, and relationships between constructs and themes identified. All codes were organized through categories of disease (asthma, cardiovascular, diabetes plus multiple NCDs) and household wealth level (wealth groups 1–3, with 1 being the lowest and 3 the highest) and themes of affordability and availability. Since there was only one patient with diabetes, we included this patient in the group of other patients with multiple NCDs, most of them had diabetes plus hypertension, asthma or both. Wealth quintiles were merged, and three wealth group formed based on the similarities in their findings: Wealth Group 1 corresponded with wealth quintiles 1 and 2, Wealth Group 2 corresponded with wealth quintile 3 and 4, and Wealth Group 3 corresponded with wealth quintile 5. The wealth quantiles were originally constructed by researchers based on an asset index. Researchers read and performed a selective analysis leading to identification of salient themes.

### Ethical considerations

The protocol was approved by the Institutional Review Boards at Maseno University in.

Kenya and at Boston University Medical Center, USA. Written informed consent was obtained for all participants at the start of the study. All participants in the household surveys were compensated with 50 Kenyan Shillings (USD $0.50), an amount deemed appropriate by the local Institutional Review Boards and local counterparts. All methods were performed in accordance with the relevant guidelines and regulations.

## Results

### Demographic characteristics

Table 1 summarizes the sample socio-demographic characteristics. The large majority were female, married or living together, one fifth had no schooling and one fifth had more than secondary school education. A larger proportion of patients with asthma had no education and belonged to the lowest wealth group.

**Table 1:** Sample characteristics

	Total (*N* = 43)	Hypertension (*N* = 23)	Asthma (*N* = 10)	Diabetes or other multiple NCDs (*N* = 10)
Age	62.4 (13.9)	66.0 (14.2)	55.3 (16.4)	61.5 (7.6)
Gender:				
Male	10 (23 %)	8 (35 %)	1 (10 %)	1 (11 %)
Female	33 (77 %)	15 (65 %)	9 (90 %)	9 (90 %)
Marital Status:				
Single	2 (5 %)	1 (4 %)	1 (10 %)	0 (0 %)
Married or living together	29 (67 %)	18 (78 %)	5 (50 %)	6 (60 %)
Divorced or separated	1 (2 %)	0 (0 %)	0 (0 %)	1 (10 %)
Widowed	11(26 %)	4 (17 %)	4 (40 %)	3 (3 %)
Education:				
Preschool (less than 1 year completed)/NONE	8 (19 %)	6 (26 %)	1 (10 %)	1 (10 %)
Primary School (not completed)	11 (26 %)	4 (17 %)	6 (60 %)	1 (10 %)
Primary school	7 (16 %)	4 (17 %)	1 (10 %)	2 (20 %)
Secondary school	10 (23 %)	6 (26 %)	1 (10 %)	3 (30 %)
Higher than secondary school	7 (16 %)	3 (13 %)	1 (10 %)	3 (30 %)
Vocational School (Post primary)	0 (0 %)	0 (0 %)	0 (0 %)	0 (0 %)
household size - adults and children	4.3 (3.0)	4.3 (3.2)	3.9 (2.7)	5 (3.0)
Wealth Groups				
Lowest	9 (21 %)	5(22 %)	4 (40 %)	0 (0 %)
Middle	20 (47 %)	14 (61 %)	3 (30 %)	3 (30 %)
Highest	14 (33 %)	4 (17 %)	3 (30 %)	7 (70 %)

Note: Continuous variables reported as mean (SD) and categorical variables reported as N (%); no household size reported for 4 households due to missing data

The main themes that emerged from the data include: coping mechanisms based on disease symptoms, tradeoffs, social capital and medicine management in health systems.

### Theme 1: Coping mechanisms based on diseases symptoms

Participants within each disease category reported experiencing life-threatening symptoms that required emergency attention, and non-urgent symptoms that required attention but allowed for some delay. Respondents’ sense of urgency regarding their symptoms and proximity to accessing medicine determined their coping strategies.

#### Coping with urgent symptoms

##### Asthma

When patients with asthma were unable to get their medicines, they described that staying without medicines could lead to an onset of severe symptoms. This included difficulty breathing, feelings of a congested chest, and challenges in completing simple tasks such as walking, standing up for short periods of time, and working. If symptoms escalated, patients and family caretakers felt an immediate sense of urgency to find ways of getting the medicines. One of the options considered the fastest choice of treatment and relief was to use emergency inpatient services or low-cost injections for immediate symptom relief.

“*I feel very bad because I can’t even breathe, I can’t sit, I can’t even walk or to stand alone I can’t. I must feel bad, at that time I feel bad, I wish to get the medicine take it quickly then I feel better*.” *– Kakamega, Asthma*.

##### Multiple NCDs (Asthma, Diabetes, Cardiovascular Disease)

Patients who had multiple NCD’s described intensified symptoms associated with lack of access to medicines. One patient described their mother’s abrupt hospitalization when she was unable to access medicines briefly for two or three days, emphasizing the critical impact of lack of medication access and symptom acceleration.

*“You see now my mother is sick and she needs drugs, when there is no drug it really puts her down even there is a day, she didn’t get the drugs until she fainted in the house just because of failing to get the drugs. Even the doctor… I was even carried in a vehicle. She had to be admitted that day.” -Samburu, Multiple Diseases*.

Another patient with multiple NCDs explained that even short lapses in medication could be life threatening or affect the progression of any one of their conditions.

*“Because when I developed this condition, the doctor informed me that I will have to be on medication my entire life. Therefore, he told me that if I stop taking medication for diabetes and as a result, the sugar levels go up or down… sometimes it may refuse to stabilize and you might die. For hypertension, if you stop the medication, a spike in pressure might result in a stroke and that might kill you. If it is asthma, if you stop medication and get the asthmatic attack it becomes difficult to clear. As a result, you might be hospitalized otherwise you might die.” -Embu, Multiple Diseases*.

#### Coping with non‐urgent symptoms

##### Asthma

Patients with non-urgent asthma symptoms used various coping methods when medicines were unavailable. Several patients withheld medicine purchases, avoided work so as to not agitate symptoms or used traditional and herbal remedies to delay symptoms from becoming severe. To seek medicines at the most affordable price, patients visited multiple public and private facilities. Some patients sent relatives to obtain medicines or asked people living in neighboring cities to send medicines via mail or by car. This was particularly relevant to asthma patients who were unable to travel long distances and were also aware of the volatility of their health condition.

*“I started using traditional remedies but that didn’t help me because asthma is something that comes suddenly and attacks you when you are seated and makes you fall … you need something quick like an inhaler to inhale or injection.”* – *Kwale, Asthma*.

##### Cardiovascular Disease

Patients with cardiovascular disease in need of non-urgent care delayed purchasing medicines for three to four days and sometimes up to two weeks at a time. This allowed some patients to purchase medicines in a larger city, which increased the chances of finding medicines at a lower price. This was an important coping strategy since patients voiced cost burdens associated with inconsistent availability and the progressive depletion of wealth and inability to work due to their chronic disease management. Although patients were able to get medicines from larger cities, they acknowledged the challenges associated with using out of pocket funds and additional indirect costs including transportation fees and selling family assets such as livestock to obtain cash. Patients also struggled with how often they could reach out to friends, family members and relationships with staff at chemists to borrow funds or ask for medicines on credit. Patients understood that their chronic conditions could deplete social and monetary capital over time which added a component of emotional stress related to managing health while also searching for sources of funds and the most cost-effective medicine purchases.

*“Once they miss in the hospital, you have to buy the medicines…Somebody is advised to collect them the next day. But the process to collect the next day, one prefers to buy instead of wasting fare going the next day*.” -*Embu, Cardiovascular Disease*.

##### Multiple NCDs

Under non-urgent circumstances, patients with multiple NCDs voiced the significant financial burdens associated with purchasing multiple medications to treat their NCDs. Therefore, to make the most economic choice, patients preferred to visit public health facilities and use insurance as their primary mode of payment. Patients felt that delaying medicine purchases was worthwhile to maximize insurance benefits and avoid out of pocket costs. Under non-urgent symptoms, patients regularly went to public health facilities to get tested for blood pressure and sugar levels. The frequency at which patients monitored health conditions determined opportunities to look for and receive medicines and potentially influenced the perception of availability at health facilities. In the face of unavailability, patients searched for medicines in private facilities and voiced that price was of utmost concern. This resulted in patients searching for medicines in locations with multiple chemists at different price points.

### Theme 2: Wealth and tradeoffs to overcome barriers of access to NCD medicines

Respondents with NCDs across the three wealth levels coped with frequent medicine stock outs in the public facilities by purchasing from chemists or private facilities. In order to afford NCD medicines from the private sector, household members described a range of tradeoffs, such as: forgoing essential items, selling assets, and spending their savings. Households, regardless of wealth group, described significant tradeoffs in order to afford medicine. However, poorer households in groups 1 and 2 frequently reported larger sacrifices, such as forgoing basic food or depleting household assets, compared to their counterparts in wealth group 3, where these tradeoffs were less common.

#### Forgoing essential items

Respondents across all wealth levels reported forgoing essential items in order to cover the cost of NCD medicines. Items deemed essential were relative to their wealth group, all of which had implications for the household livelihoods. For example, the lowest wealth level frequently reported items such as food, hygiene products, and clothing:

“*sometimes you might go with your program, you find something is missing you say better I miss food today I get this, I better lack this cloth I get this one” -*Narok, Wealth Group 1.

“*Sometimes even when I don’t have bathing soap and I don’t have money I forego buying soap so that I can buy medicine.”—Embu, Wealth Group 1*.

Some households in the second level wealth group described frequently forgoing certain types of food, and most described items such as sugar, school fees, and clothing.

*“That whole year, we have had to stay without using the basics, you are forced to forgo one thing to be able to get the drugs. Like if you were to buy beans, you are forced to cook food without beans”–Nyeri, Wealth Group 2*.

*“It has taken us backward so much, we don’t pay school fees and the issue is the chest, and food you also deny yourselves you don’t buy because first there is the drugs.” –Kwale, Wealth Group 2*.

Households in the upper wealth group mentioned forgoing items with less frequency than wealth groups 1–2, however clothing, school fees, ice cream, meat, and petrol were all identified.

*“before, we used to use things like ice cream but right now, we cannot…We used to eat chicken, now we do not,…What really affected us most is movement by use of a vehicle…Petrol is expensive therefore, we decided instead of moving, let us set aside the money. So we took our money to buy to drugs.”*—Embu, Wealth Group 3.

#### Reduction in wealth and assets

Respondents at all wealth levels list selling a range of assets in order to cover the cost of their NCD medicine; chickens, goats, and land were sold at various times. Households in the mid- and lower-wealth groups frequently reported selling assets in order to cover the cost of NCD medicine; whereas this coping mechanism appeared with less frequency in households in the upper wealth group.

*“I have to sell the chicken cheaply so that I can get medicines, rearing chicken you can only do that if you are not sick and are focused, if you have the chicken you will sell it so you can buy a goat but if I am sick I am not able to buy the goat but buy the medicines.” –Embu, Wealth Group 1*.

*“Wealth has decreased, truly wealth has decreased because of the way the illness affects mother, we need to sell our wealth, to use a lot of money and even go into debts because of her condition.”—Samburu, Wealth Group 2*.

*“Yes, I had a car and I sold. I had a cow and I sold. So I have got just one cow and two calves.”—Nyeri, Wealth Group 3*.

### Theme 3: Using social capital for financial and social support

Social capital has been defined as “the informal and organized reciprocal networks of trust and norms embedded in social organization of communities –with social institutions both hierarchical and horizontal in structure” [22, 23]. The respondents relied on their social capital through connections with friends, relatives and co-workers to access NCD medicines, ranging from borrowing funds, purchasing medicines on credit, or being granted reduced prices. However, households at the lowest wealth levels had less leverage to lean on social networks to gain access to NCD medicines. Their social networks were unable to provide comparative financial or social support in accessing medicine as compared to households in the mid-and highest wealth group. The following sub-themes explore the relationship between social capital and wealth quintile.

#### Borrowing money & assets

Households from the lowest wealth group frequently described trying to borrow money from their social networks, often without success. They were expected to repay the debt, which added an additional financial burden in addition to regular payments for NCD medicine. For those in the lowest wealth group, borrowing money could lead to impoverishment because households had to consistently pay debts, making it difficult to save or invest meaningfully. Respondents in the middle and highest wealth groups frequently received financial assistance from their children without problem.

*“I usually feel bad because…I usually don’t have money, so when they tell me to go buy I…I have to start thinking of going to [mentions a name] so that I can borrow money to buy this drug so that I don’t go disturb people at home, so I just go somewhere and borrow so that even if I get one packet so that I can go back with it at home.”* -Embu, Wealth Group 1.

This financial assistance may be a relief for patients but may have implications on their children’s wealth, resulting in an intergenerational depletion of household wealth and assets.

*“Initially, like now we have livestock, goats there are times mother is so ill that we are forced to sell a goat to be able to cater for her medication. If my brother doesn’t have money here we are forced to send a goat to be sold to reduce the bill, sometimes mother has money in her account we go into the mothers account and withdraw some money just for the illness. This disease has made mother to spend her more than usual.” –Samburu, Wealth Group 2*.

#### Personal relationships with chemist/facility staff

Respondents in the middle and highest wealth group also reported that their professional or personal connections to chemist and facility staff facilitated their ability to access NCD medicines at reduced or free prices. Very few examples of this were found during interviews with patients in the lowest wealth group.

*“…there is a drug for her for asthma that she normally has, now if you go, there is a chemist around here and she and my mother know each other so she will just give you, she will sell it to you cheaply and not expensive.”* Samburu, Wealth Group 3.

#### Alternative payment structure

Multiple patients described being granted flexibility in payment structure. It was not clear if this was due to the patient’s personal relationship to staff at the facility, or if it is simply facility policy and that these patients know where to go, in order to obtain such flexibility. Regardless, these types of experiences were often described in the lowest wealth groups.

*“when we go there and we don’t have money, we tell them we will pay later they trust us we will pay when we get money.”* -Samburu, Wealth Group 2.

### Theme 4: Health insurance does not guarantee access to NCD medicines

One salient challenge patients mentioned was the execution of the National Hospital Insurance Fund (NHIF). No patients in lowest wealth group used health insurance to help pay for medicine, whereas patients in the middle and upper wealth group frequently reported using health insurance.

Some patients required multiple medicines and purchased NHIF insurance to offset monthly costs. However, the value of the NHIF was questioned when patients arrived at the public facility to find their medicines unavailable. Often described as a “double-cost,” patients who purchased health insurance expressed their frustration paying for medicines at private facilities because the public facilities were out of stock.

*“[Y]ou are disappointed, you just see how you have wasted money and then there is no drug, you will be forced again to buy it which is a double cost.” -Kakamega, Cardiovascular Disease*.

There were exceptions where patients felt that NHIF had assisted in the purchase of medicines. Some patients observed increased access to free blood pressure and diabetes medications, compared to previous years where only half of prescribed medications were available. Ultimately, many patients accepted their health circumstances and the unavailability of medicines, and planned for necessary funds to afford both direct and indirect costs.

*“I know that I use fifteen thousand per month on drugs …therefore, if I find them at …I am grateful. And, if they are unavailable, I keep the money knowing that next month… all that is because of uncertainty of whether the drugs will be available or not. I, however, make sure that I have the money…” -* Embu, Multiple NCDs.

## Discussion

This paper has illuminated how patients cope with the lack of availability and affordability of NCD medicines at the household level, categorized by disease and wealth level in eight counties in Kenya. Regardless of wealth level and disease type, most patients struggled with frequent medicine stockouts at health facilities, which forced patients to turn to the private sector in order to procure NCD medicines. The price of NCD medicines and opportunity costs were the greatest barriers to accessing medicines from chemists and other private facilities. Households made significant tradeoffs in order to cope with the high prices of medicines, often resulting in the depletion of household wealth over time. These findings suggest that NCD medicine availability and affordability remain an ongoing issue ([24].

Our study contributes to knowledge on the experiences and opinions of patients on how they cope with access to NCD medicines. Although strategies such as borrowing funds, forgoing essential items, selling off assets, and relying on social networks to procure medicines have all been documented in other studies [7, 10–12].

Our study provides insights into coping with a lack of availability and unaffordability of NCD medicines across three prevalent NCDs and wealth levels.

Firstly, coping with unavailability and unaffordability of NCD medicines varied among patients depending on the disease. Differences in disease progression and symptom manifestations suggest a temporal component that determine coping strategies and economic pathways. Secondly, while the findings are consistent with existing literature that NCD medicines pose a significant financial burden to Kenyan households [7–9] they add a noteworthy nuance: while all wealth levels made substantial tradeoffs in order to access NCD medicines, the tradeoffs made by households within lower wealth levels had long term repercussions on household wealth and stability. Coping with the high cost of NCD medicine by selling certain assets precipitated a cascade of additional financial burdens or loss of livelihoods. For example, one of the most common liquidated assets mentioned was livestock. In addition to providing useful commodities, such as eggs, milk, or meat that the families may depend on for daily living, livestock represent an investment in future wealth and earnings. Selling these can lead to significant loss of livelihoods in the long-term and contribute to intergenerational poverty.

Thirdly, although similar studies have found that patients rely on social networks to facilitate medicine access [7, 10], our results show that the leverage offered by these networks was different across wealth categories. Households from lower wealth levels were less likely to rely on access social networks for financial support, reduced prices, or notifications of medicine stocks. People in the middle and upper wealth levels were able to use their social capital to establish relationships and trust with private chemist and health facility staff, which further rewarded their access to NCD medicine.

Finally, the introduction of a national insurance system has been traditionally viewed as a financial asset to NCD patients (Kankeu et al., 2013). We found that patients with cardiovascular disease and multiple NCDs with multiple medicines relied on the insurance system as a means to offset high monthly medication costs. However, inconsistent public sector supply resulted in patients not receiving medicines free of charge at the point of care even though they were included in the insurance benefit package. Patients therefore, faced a double financial burden of paying for health insurance premiums plus their monthly medicines. Scaling up the roll-out of health insurance will require reliable public sector supply of medicines that are included in the insurance benefit package in order to protect patients from financial expenses on medicines.

### Study limitations

There were some limitations to these findings. The original study design addressed questions regarding the impact of the Novartis Access programs on the availability and affordability of NCD medicines. Categorizing by disease and wealth group was part of a secondary analysis which limits the generalizability of the results. Future studies could consider the number and types of individuals that patients rely on within social networks for assistance. Additional research questions related to disease progression could clarify the progression and long-term impact of medicine access programs. Additionally, sampling across wealth and disease groups could ensure that the results are representative of the specific populations of interest.

### Recommendations

Across all disease types, patients voiced the additional costs and burdens of searching within public and private facilities for medicines. Public and private facilities facing inconsistent medicine supply could benefit from a transparent supply chain system where stock level information is made public to patients, health providers, and administrators via SMS or other available mobile technologies. Such a system would notify patients about medicine stock prior to traveling to the facility, saving patients the experience of spending resources and time on travel to find medicines unavailable. Other strategies to promote transparency could include top down methods of information access, where clinicians and or community health workers (CHW) regularly visit NCD households to provide support, education and information about available supplies. NCD care studies using CHW show the benefits of this direct access, and how this model could address NCD education and holistic aspects of health in addition to NCD medicine access [25].

Additional studies have addressed expansion of insurance programs to cover the cost of medicines at private facilities as well as low enrollment rates amongst rural communities within Kenya [12, 26]. Currently, private chemists do not accept insurance, which can serve to increase cost barriers to accessing NCD medicines [27]. Expanding insurance coverage and reimbursements within private facilities could reduce financial burdens and minimize the indirect costs associated with inconsistent supplies at public facilities. Our results also suggest that patients of lowest wealth group did not utilize insurance as often as highest wealth group. Therefore, the implementation of insurance programs must target the lowest wealth groups. Insurance should be expanded in parallel to the improvement of medicine supply chains, and insurance coverage across public and private facilities.

Our findings from wealth groups suggest that individuals who possess a larger social network and existing financial assets are better positioned to cope with NCD medicine management. Individuals who reached out to family members and owned larger financial assets typically could withstand the long-term financial costs of medicines. Therefore, strengthening social networks could specifically prioritize low income patients. Future economic studies of household assets sold to purchase medicines, the expenditure on medicines and its impact on impoverishment would be valuable.

Lastly, the theme of emotional stress related to chronic disease management surfaced throughout the interviews. While this was not the focus of this study, this area warrants further exploration, taking into consideration a more expansive definition of health such as the WHO’s which incorporates the concept of overall wellbeing into its definition of health [28]. This is particularly, among patients diagnosed with cardiovascular disease and multiple NCDs diagnosed at an early stage of life. These patients voiced the emotional toll related to procuring NCD medicines and the subsequent impact on self-identity and community engagement. And lastly, longitudinal studies could illuminate the progression of social and financial impact of NCD medicine access.

## Conclusions

The type of coping strategies to access barriers differed by non-communicable disease and wealth group. Although Kenya has made important steps to address access to NCD medicines challenges, prioritizing enrollment of low wealth households in county health insurance programs and ensuring continuous availability of essential NCD medicines at public health facilities close to the patient homes could significantly improve access.

## Data Availability

The datasets generated and analyzed during the current study are not publicly available due protection of the confidentiality of the participants. The respective institutional review boards have not provided authorization to make the datasets available. For further questions on the availability of the data please contact the corresponding author.
